# ABO blood group in Malagasy patients with cancer: which group predominates?

**DOI:** 10.11604/pamj.2019.32.73.17994

**Published:** 2019-02-12

**Authors:** Nomeharisoa Rodrigue Emile Hasiniatsy, Elodie Emile Batavisoaniatsy, Valéry Refeno, Mamy Andriambololona, Andriatsihoarana Voahary Nasandratriniavo Ramahandrisoa, Florine Rafaramino

**Affiliations:** 1 Oncologie Médicale, Faculté de Médecine d’Antananarivo, Antananarivo, 101, Madagascar; 2 Immunologie, Allergologie, Faculté de Médecine d’Antananarivo, Antananarivo, 101, Madagascar; 3 Oncologie Médicale, Faculté de Médecine d’Antananarivo, Antananarivo, 101, Madagascar; 4 Biologie Médicale, Faculté de Médecine d’Antananarivo, Antananarivo, 101, Madagascar; 5 Oncologie Médicale et Radiothérapie, Faculté de Médecine d’Antananarivo, Antananarivo, 101, Madagascar

**Keywords:** ABO blood groups, cancer, Madagascar, oncology

## Abstract

The blood group of Malagasy patients with cancer have never been the subject of previous publications. Our objective was to determine the blood group of Malagasy patients with cancer followed in the Medical Oncology Unit of the Soavinandriana Teaching Hospital, Antananarivo. This was a one-year retrospective study (November 2012 to October 2013) in patients over the age of 15 with histological or pathological evidence of their cancer. One hundred and thirty of the 258 patients identified had an ABO blood group determination (50.39%). Among these 130 patients, 114 patients (87.69%) had solid tumors and 16 patients (12.31%) had hematologic malignancies. Thirty seven (28.49%) patients were transfused and 93 (71.54%) not transfused. There were 57 men and 73 women (sex ratio = 0.78), the average age was 55.11 +/- 14.76 years. With regard to their blood group, 52 patients (40%) were blood group B, 44 (33.84%) group O, 27 (20.76%) group A and 7 (5.38%) group AB. The order of blood group frequency of cancer patients in our series differs from other studies. This study has allowed us to know the proportion of each blood group in our Unit and thus help us in the management of stocks of labile blood products in our hospital.

## Letter to the editors

Few studies on the Malagasy blood group have been conducted in the general population and among certain categories of population [1, 2]. For the cancer, although the association between ABO blood groups and the occurrence of cancer has been reported in the literature [3-5], no study on this subject has been conducted in Madagascar. To our knowledge, there is no known repartition of the blood group of Malagasy cancer patients published. This study aimed to determine the ABO Rhesus blood group of cancer patients in the Medical Oncology Unit of the Soavinandriana Hospital Center (CENHOSOA) in Antananarivo, Madagascar. We report here the results of a year of transversal retrospective study (November 2012 to October 2013) within the CENHOSOA Medical Oncology Unit. We have described the blood groups according to the ABO system of patients over 15 years old, with histological or cytological proven cancer, recorded in this unit. The data was collected on Excel 2003^®^ software. One hundred and thirty of the 258 patients identified had an ABO blood group determination (50.39%). Among these 130 patients, 114 patients (87.69%) had solid tumors and 16 patients (12.31%) had hematologic malignancies. Thirty seven (28.49%) patients were transfused and 93 (71.54%) not transfused. There were 57 men and 73 women (sex ratio = 0.78), the average age was 55.11 +/- 14.76 years. With regard to their blood group, 52 patients (40%) were blood group B, 44 (33.84%) group O, 27 (20.76%) group A and 7 (5.38%) group AB (Table 1).

**Table 1:** Distribution of cancer patients followed in the Medical Oncology Unit of the Centre Hospitalier de Soavinandriana (November 2012-October 2013) according to their ABO blood group

ABO blood group	Number	Frequency (%)
**A**	27	20.8
**B**	52	40.0
**O**	44	33.8
**AB**	7	5.4
**Total**	130	100.0

This is the first ABO blood group study in Malagasy patients with cancer. It allow us determine the blood groups' repartitions of some Malagasy patients suffering from cancer. Although in the Malagasy population, knowing the blood group is not common [1, 2], the blood group of the half of our patients was known. The order of blood group frequency of cancer patients in our series differs from the blood group studies performed in young recruits of the Moramanga gendarmerie school in 1969 (out of 2411 men) and in patients who came to the Androva emergency room (Mahajanga) in 2010 (out of 51 patients). Our study suggests that group B (40%) is the main blood group found in cancer patients treated in the CENHOSOA Medical Oncology Unit. In fact, this blood group does not exceed the percentage of subjects of group O (43.8% against 29.3%) in the Mayoux study in 1969 [1]. In the Emergency Department of Androva Hospital (Mahajanga) group B came third (17.6% of patients) after group O (47.1%) and group A (27.5%) [2]. These results should be interpreted with caution as our study, while being retrospective, was performed clinically and the blood group was the one reported in the patient's chart. The increasing risk by the blood group B or protection by the other blood groups regarding the cancer could not be verified in our study because of the absence of non-cancerous controls. Nevertheless, these results will serve to make aware the blood bank of our hospital on the management of their reserve. In addition, they encourage us to perform a case-control study comparing cancer patients with the general population. Indeed, this role of group B as a risk factor for cancer is rarely reported in the literature [3-5]. Although people in group A would be at greater risk of developing cancer compared to those in non-A groups and blood group O would be associated with a decreased risk of cancer development in most of the literature data [3, 4], some population such as India where the 42.05% of cancer patients are group B, would be an exception [5]. Thus, the association between group B and cancer is to be verified among Malagasy to assess our results.

## Conclusion

The order of blood group frequency of cancer patients in our series differs from other studies. The possible biases of our study remind us of the need to carry out studies determining the association between blood groups ABO and the risk of developing cancers in our country. Nevertheless, this study has allowed us to know the proportion of each blood group in our Unit and thus help us in the management of stocks of labile blood products and awareness of donations of blood in our hospital.

## Competing interests

The authors declare no competing interests.
